# The neural correlates of biomechanical constraints in hand laterality judgment task performed from other person’s perspective: A near-infrared spectroscopy study

**DOI:** 10.1371/journal.pone.0183818

**Published:** 2017-09-01

**Authors:** Shuang Meng, Misato Oi, Godai Saito, Hirofumi Saito

**Affiliations:** 1 Department of Cognitive and Psychological Science, Graduate School of Informatics, Nagoya University, Nagoya, Japan; 2 Faculty of Arts and Science, Kyushu University, Fukuoka, Japan; 3 Department of Psychology, Graduate School of Arts and Letters, Tohoku University, Sendai, Japan; Tokai University, JAPAN

## Abstract

Previous studies, mainly using a first-person perspective (1PP), have shown that the judgments of the hand laterality judgment (HLJ) task are dependent on biomechanical constraints (BC). Specifically, differing reaction times (RT) for hand pictures rotated medially or laterally around the mid sagittal plane are attributed to the BC effect on motor imagery. In contrast, we investigated whether the HLJ task is also subject to BC when performed from a third-person perspective (3PP) as well as 1PP using near-infrared spectroscopy (NIRS) to measure the brain activity of prefrontal cortex (PFC) in right-handed participants assigned to 1PP or 3PP groups. The 1PP group judged whether a presented hand was their *own* left or right hand, and the 3PP group whether it was the *other’s* left or right hand. Using their HLJ task error rates, the 1PP and 3PP groups were subdivided into an Error Group (EG) and No Error Group (NEG). For the 1PP group, both EG and NEG showed a significant Hand Laterality × Orientation interaction for RT, indicating the BC effect on motor imagery. For the 3PP group, however, neither EG nor NEG showed the interaction, even though EG showed a significantly longer RT than NEG. These results suggest that the 3PP EG appropriately followed the 3PP task instruction, while the NEG might have taken 1PP. However, the 3PP EG NIRS profile of left PFC showed a significant Hand Laterality × Orientation interaction, while the 1PP EG did not. More noteworthy is that the left PFC activation of EG showed an interaction between the 1PP and 3PP groups when the left hand was presented. Furthermore, in the NEG, the PFC activation was not influenced by the BC in either the 1PP or 3PP condition. These results indicate that BC interferes with the HLJ task performed from the 1PP and 3PP.

## Introduction

Shepard and Metzler [1] demonstrated that mental rotation (MR) is the cognitive process of imaging an object rotating from one orientation to another. They reported that the reaction time (RT) required to judge whether the pairs of two three-dimensional cubes were the same increased linearly with greater angular disparity between the two cubes. This result suggests that participants mentally rotate one of the objects into congruence with the other before comparing the two objects, i.e., imagined object rotation depends on the object-relative reference frame (i.e., *allocentric* perspective), which specifies the location of an object’s parts with respect to each other [2]. The linearly increased RT in the MR task has been confirmed in a number of studies using *objects* as stimuli, for example, alphanumeric characters[3], letter-like characters [4], and two-dimensional unfamiliar shapes [5], but not in the studies using body parts such as hands, as stimuli [6, 7].

For instance, the hand laterality judgment (HLJ) task is usually used to examine the MR of *body* parts in which participants judge whether a *hand* presented at various orientations is a left or right hand. Several studies found that “The time required for the laterality (left or right) of a hand presented in different views and angular orientations is similar to the time taken to execute a corresponding movement” [6–10]. These results suggest that the “Hand stimuli are automatically coded with respect to an *egocentric* (internal) perspective (i.e., according to the viewpoint consistent with looking at one’s own body)” [11]. That is, “To perform the HLJ task, participants mentally rotate their upper limbs toward the position of the displayed stimulus in a way that is consistent with the biomechanical constraints (BC) underlying the actual movement” [9]. These results of BC effect were interpreted as indicating that motor imagery plays a critical role in the HLJ task, while visual imagery plays a dominant role in the MR of an object task [12].

The above-cited behavioral results indicate that two distinct mechanisms may be used in the MR task, that is, the MR may be accomplished by taking a reference frame from the object itself (i.e., *allocentric* perspective) or the viewer’s (i.e., *egocentric* perspective) [13]. Furthermore, previous studies found that although the MR of an *object* is automatically performed from an *allocentric* perspective [1–5, 12], it might also be performed from an egocentric perspective [14]. Kosslyn et al. [14] measured the brain activity of MR of 3D cubes when participants were using internal (egocentric) or external (allocentric) strategies in a positron emission tomography (PET) study. The behavioral results showed that the RT increased with the amount of orientation and there was no difference between the internal and external conditions, indicating that the MR of an object using the internal or external strategy was not modulated by BC. The imaging profiles showed that the motor areas were activated when participants were instructed to use an internal strategy, while no motor activation was present when participants were instructed to use an external strategy. Taken together, these results suggest that there are two ways to imagine an object rotating and participants may adopt one or the other strategy according to the experimental requirement.

Analogously, although MR of *body parts* is automatically conducted by taking an *egocentric* perspective, it might also be performed from an allocentric perspective [11, 15–16]. Previous studies reported that the *hand orientation*, the *context features* (e.g., “showing a hand stimulus within a human silhouette” [11]), and *instructions* of the experiment may induce a shift from an egocentric perspective to an allocentric perspective during the task of MR of body parts [11, 15–16]. Furthermore, it has been shown that the MR of body parts conducted from an egocentric perspective was subject to BC [6–10]. However, it is still unclear whether the MR of body parts conducted from an allocentric perspective was influenced by BC or not. In the present study, we explicitly asked the participants to judge the laterality of the hand from their *own* perspective or the *other* person’s perspective to investigate the BC effect in the egocentric and allocentric perspectives.

To test whether BC is subject to both 1PP (first-person perspective) and 3PP (third-person perspective) in the motor imagery task, Anquetil and his colleague [17–18] compared the RT difference between *easy* (participants *can* comfortably grasp the cylinder in “45°”) and *difficult* (participants *cannot* comfortably grasp the cylinder in “0°”) orientations with their right hand during a perspective taking task (1PP vs. 3PP). Perspective taking requires the participant to transform the egocentric view from 1PP (Is the line on *your* right or left?) to 3PP (Is the line on *his* right or left?) in which mental states are ascribed to someone else [19]. Anquetil and his colleague [17–18] reported that the motor imagery task from both the 1PP and 3PP are modulated by BC. However, it is not clear whether participants in their studies might perform the motor imagery task from the 1PP instead of the specified 3PP condition.

In order to investigate whether a self-generated and an observed action shared the same representations or not, Anquetil and his colleague [17] asked the participants to imagine themselves performing the action of grasping an upright cylinder with their “right” hands (self-generated action) during the 1PP condition, and imagine another person facing them performing the same action (observed action) during the 3PP condition. The results showed that there was no difference between the time to complete the movement from 1PP and 3PP. They suggested that “Participants in fact performed the same action from the two perspectives, as if they had mentally rotated themselves so as to superimpose with the virtual subject facing them”. More importantly, compared to grasping the cylinder with an *easy* orientation (45°), the time for grasping the cylinder with a *difficult* orientation (0°) was longer. The result indicates that mentally simulating a grasping movement from both the 1PP and 3PP conformed to the same BC. In their following study [18], they reviewed the study of Anquetil and Jeannerod [17] and proposed a self-other differentiation model consisted of two steps: “I first displace myself at the location of the person I observe in order to specify her/his location in space. Then, I simulate the action I observe from that person in order to understand what she/he is doing. Assuming that the two operations are more or less synchronous, the action that I simulate is automatically attributed to the person I observe, not to myself” [18]. Based on the self-other differentiation model, 3PP processing should result in a longer RT than 1PP processing, as the 3PP processing requires multiple stages of processing while 1PP processing does not. Actually, several studies have shown that participants’ RTs in the 3PP condition were longer than those in the 1PP condition [11, 15, 20].

In order to investigate the BC effect in 3PP using a HLJ task, we measured the brain activation of the prefrontal cortex (PFC), which has been reported to be involved in perspective taking and motor imagery [20]. Mazzarella et al. [20] investigated the brain regions engaged in egocentric (1PP) and allocentric (3PP) tasks in an fMRI study. They required participants to decide whether an orange on a round table was on the *participants’* left or right in the egocentric (1PP) task, and whether the same orange was on the *actor’s* left or right in the allocentric (3PP) task. During the experiment, participants were presented with a photo of the actor standing around the table at eight different orientations. The results showed that the activation of middle PFC (mPFC) increased with the increment of angular distance and the actor’s orientation in the allocentric task. The authors suggested that these results demonstrated that the engagement of mPFC is related to the varying demands placed on mental self-rotation required during perspective taking. In contrast, the activation of right inferior frontal gyrus (IFG) increased with the increment of angular distance and the actor’s orientation. They interpreted that the right IFG was engaged in an egocentric task which requires inhibition of automatic consideration of the actor’s perspective.

Mazzarella et al. [20] demonstrated that the mPFC is involved in the 3PP processing. Their interpretation was that mental self-rotation during perspective taking has similar properties to motor imagery. That is, one has to imagine the movement of one’s own body. Accordingly, the mPFC is related to both motor imagery and perspective taking.

In the present study, we measured the PFC activation by a two-channel wireless portable near-infrared spectroscopy (NIRS) device (pocket NIRS) in order to examine whether PFC is involved in the BC effect in the HLJ task performed from the 1PP and 3PP. Compared with fMRI or PET (Positron Emission Tomography), NIRS has relatively fewer physical constraints, permitting assessments of tasks in natural settings [21]. During NIRS measurement, the subjects wear a fiber holder of probes allowing them much greater freedom of movement, while during the fMRI or PET measurement, the participants lie down in the scanner and their movements are relatively restricted. Liu et al. [22] have demonstrated that the pocket NIRS may effectively measure the PFC activation.

The purpose of the present study was to examine whether BC function as a negative regulator in 3PP as well as in 1PP, using a HLJ task and a character rotation task. To test the research question, it is essential that the participants precisely follow the task demand of the 1PP and 3PP conditions. To achieve the goal in the present study, we adopted two experimental operations concerning instruction and data analysis.

Most of the previous studies using the HLJ task have been conducted without explicit instruction concerning whose hands were rotated, i.e., subjects simply judged whether the presented hand is a left or a right hand [6–9]. First, in contrast to the previous studies, in the present study the 1PP and 3PP groups were explicitly given two different instructions, i.e., the 1PP group was instructed to judge whether the presented hand is *their own* left or right hands, and the 3PP group whether it was the *others*’ hand instead of their own hand.

Second, it is important that comprehension of explicit instructions for the 1PP and 3PP groups must be tested at a performance level. To assess whether the two groups understood their instructions and followed each task demand in the 1PP and 3PP conditions, we divided all participants in the 1PP and 3PP groups into two subgroups according to their error rates in their HLJ task. In particular, participants who are instructed to take a 3PP, may be likely to adopt a strategy of 1PP due to a larger brain activity associated with higher processing demands in the 3PP condition. Beste et al. [23] separated participants into two groups (lower- and higher-performers) according to their error rates in an EEG study of MR of characters (object), and reported that the mean amplitudes of event-related potentials were larger for lower-performers than for higher-performers in an EEG study of MR of characters. To distinguish the dissociation of instruction (what was instructed) and performance (what the response was), based on the method of Beste et al. [23], we separated participants into two subgroups, according to their error rate in HLJ task. Precisely, we divided the participants in the 3PP group into two subgroups according to their error rates, and accordingly we obtained a No error-performers subgroup (NEG) and an Error-performers subgroup (EG). In the same manner, the participants in the 1PP group were divided into the two subgroups of NEG and EG. We hypothesized that the 3PP group would show longer RTs than the 1PP group, and furthermore the EG would show longer RTs than NEG and show the BC effect.

As for the RT profile in the HLJ task, on the basis of the previous studies [6–9], an interaction between hand laterality (Hand) and stimulus orientation (Orientation) on RT was postulated in the 1PP condition. Indeed, the RT for the *left* hand in the 1PP condition increases from *90°* to 270° (from *medial* to lateral rotation), while the RT for the *right* hand increases from *270°* to 90° (from *medial* to lateral rotation). In contrast to the HLJ task, we expected the character task would not have the BC effect, i.e., no interaction should be found in the character task. In the NRIS profile, we expected to obtain a same Hand × Orientation interaction on brain activation in the HLJ task, but not in the character task.

**Fig 1:**
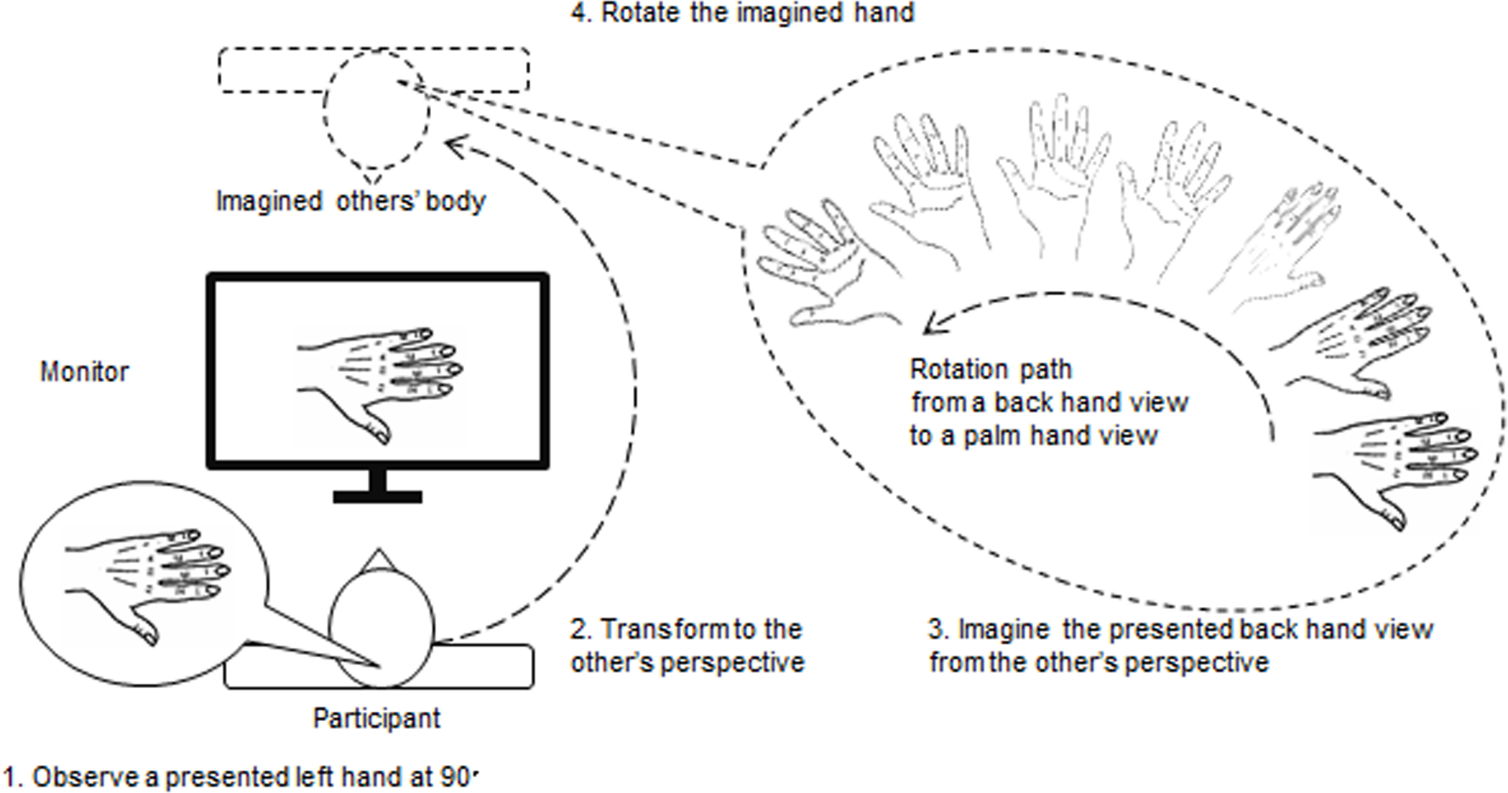
The multiple stages required for performing the HLJ task from 3PP.

On the other hand, in the 3PP condition, participants who are sitting in front of a monitor need to regard a visually presented hand on a monitor as the *others*’ hand. Fig 1 shows the multiple stages required for performing the HLJ task from a 3PP in the present study according to Jeannerod and Anquetil’s [17] self-other differentiation model. To achieve overlapping one’s own body image with the others’ body image on a monitor, the participants need to mentally rotate their own body image 180° horizontally to take a 3PP. This transforming of one’s body image to the others’ behind the monitor leads to longer RTs and PFC activation in the 3PP condition.

Furthermore, after the transformation of one’s body image, for example, to adjust the participants’ own left hand to a 90° rotated left hand on a monitor (a thumb pointing down with the *back of the hand*, see Fig 1), two ways of hand image rotation are possible in the opposite side with respect to their actual position. First, if a participant is required to mentally represent a hand from a 3PP, she/he could mentally rotate her/his body in order to place her/himself in the opposite side with respect to their actual position and maintain again a 1PP with a new “imagined” position in space, constantly keeping the same relationship with her/his hand. In this way she/he could represent the hand always as a *back*, i.e., the *observed back* of *the hand on a monitor* is always kept as the *imagined back of one’s own hand*. Second, the alternative way remains faithful to the instruction in the 3PP condition. After the transformation of one’s body image, a participant rotates (twists) their own *left* hand 180° on the Z axis to make the *palm side hand* (see, Fig 1). These two strategies share a common image transformation of one’s own body with hand(s) in a Back view but they diverge in their hand rotations (Back vs. Palm view) after the body transformation. Hereafter we call the first and second ways of hand image rotation a Back-*Back* strategy and a Back-*Palm* strategy, respectively. The Back-Palm strategy may be related to higher processing demands than the Back-Back strategy in the 3PP condition due to the relative difficulty of hand orientations and higher BC.

**Fig 2:**
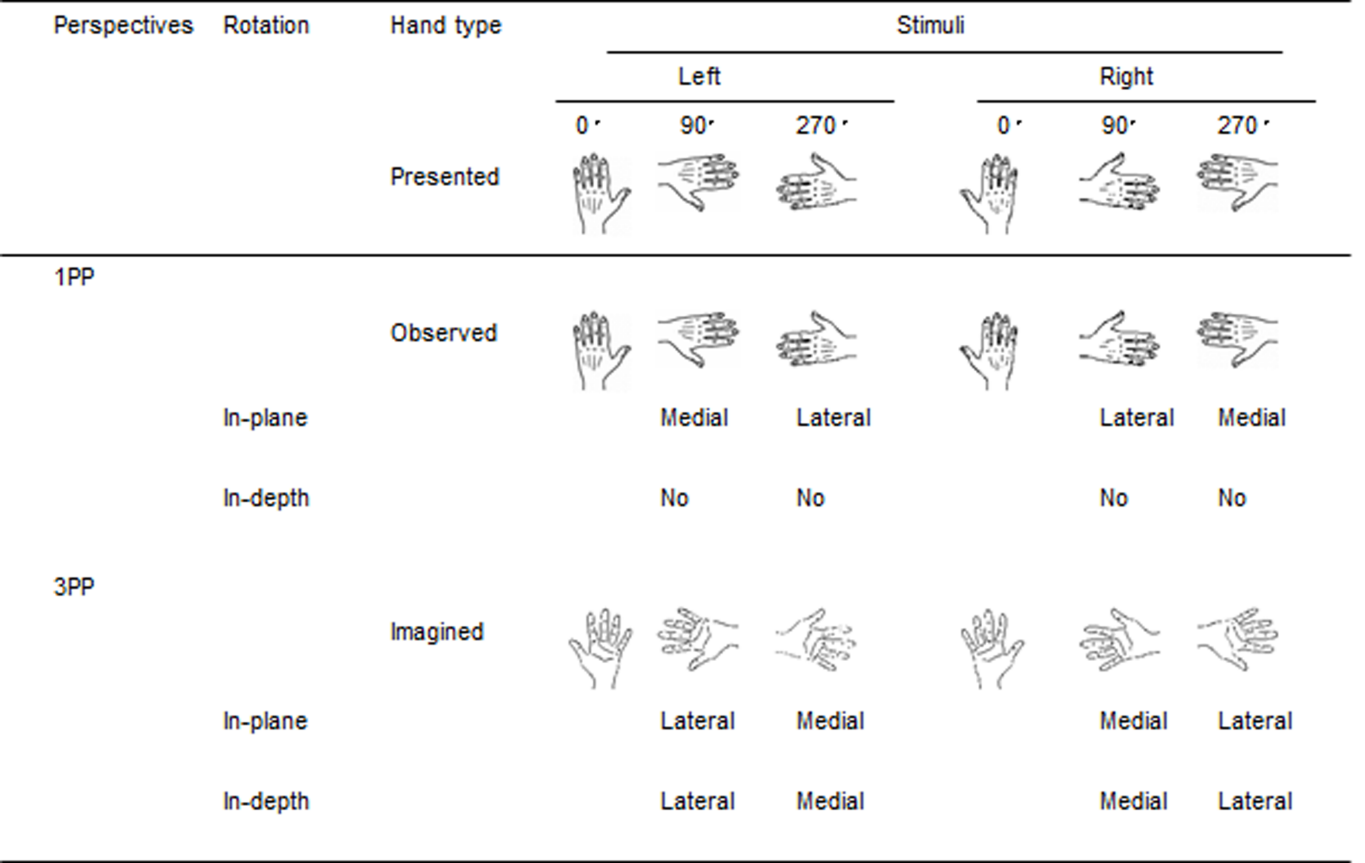
Presented and imagined hands in the 1PP and 3PP. In-plane rotation is the rotation along the x axis. In-depth rotation is the rotation along the y axis.

Previous studies have showed that a medial rotation of one’s hand is smoother than a lateral rotation due to BC of hand movements, which makes it easier to judge the hand in the HLJ task [6, 8–9, 12]. Fig 2 illustrates the *presented* hand stimuli, the *observed* hand in the 1PP condition, and the *imagined* hand in the 3PP condition using a Back-Palm strategy. If the participants in the 3PP condition take the Back-Palm strategy, to obtain the ending image of the hand in the Palm view, in a case of presented left Back view hand at 90° on a monitor, a 1PP using Back-Back strategy needs a *medial* rotation to the observed Back view hand, but a 3PP using Back-Palm strategy needs a *lateral* rotation. That is, the direction of the Palm hand view rotation (e.g., *lateral*) will be reversed to the Back hand view in the 1PP (e.g., *medial*), vice versa. Thus, we expected the interaction of Hand × Orientation in 1PP would be reversed in 3PP. That is, when we focus on a left hand, in the 1PP condition, the RT at 270° (*lateral* rotation) would be longer than at 90° (*medial* rotation), but in the 3PP condition RT at 90° (*lateral* rotation) would be longer than at 270° (*medial* rotation). Similar to the pattern of the RT profile based on the relation of medial and lateral rotations, we expected the PFC activation would show the same pattern of interactions between Hand and Orientation.

However, it is important to note that the 3PP condition requires both in-plane rotation and in-depth rotations to accomplish the task demand, while the 1PP condition requires only the in-plane rotation to accomplish the task demand. Ter Horst et al. [24] reported that “engagement in motor imagery critically depends on the number of axes of rotation of the stimulus set” in the HLJ task. Based on their results, two axes of rotation in 3PP condition would induce the BC effect in the 3PP group, if the participants exactly follow the task demand, i.e., take the Back-Palm strategy using the in-plane and in-depth rotations (see, Fig 2).

Generally, it is accepted that participants showing no errors in a task are complying with the task demand. Participants in the 3PP condition, however, may obtain shorter RT and fewer errors when they take the 1PP or a mechanical access to a memorized visual pattern without mental rotation of visually presented hand stimuli. In particular, if participants in the 3PP condition take the Back-Back strategy and may achieve a correct performance by taking 1PP without MR from 3PP, they would show the similar pattern of interactions between Hand and Orientation in the 1PP but a longer RT than the 1PP due to the transformation of their own body image in the 3PP. Taken together, if it is postulated that EG takes the Back-Palm (difficult) strategy and NEG takes the Back-Back (easy) strategy in the 3PP condition, the BC effect in the EG would be more apparent than that of NEG due to the higher processing demands in the EG.

Therefore, we hypothesized that if participants precisely follow the instruction of 1PP and 3PP in a HLJ task, their RT and brains activation to left/right hand judgment should show the BC effect. That is, there would be a similar pattern of interaction between the presented left/right hand stimuli and their orientations for both RT and brain activation, even though the pattern of interaction may be reversed between 1PP and 3PP due to the rotation path for the same hand in the 1PP and 3PP being reversed. Specifically, the interaction between hand and orientation for brain activation would be obtained to the presented left hand stimuli representing abduction of the less dexterous left hand by right-handers in the present experiment.

## Methods

### Participants

One hundred undergraduate students (96 males, 4 females; mean age: 18.560020030± 0.62 years) participated in this study for their course credit. All participants were consistent right-handers (Edinburgh Handedness Scale [25], Mean = 84.70, standard deviation (SD) = ± 12.63), who were assigned to either 1PP (N = 63) or 3PP group (N = 37). They were informed about the purpose and safety of the experiment, the written informed consent was obtained from all participants, who were native Japanese and naive to the purpose of the study. All methods were carried out in accordance with the principles and guidelines of the Declaration of Helsinki, and all experimental protocols were approved by the Institutional Review Boards of Nagoya University.

### Materials and design

The experiment consisted of two tasks: a HLJ task and a character rotation task. The experimental materials were same as in the Conson et al. [26] study. In the HLJ task, two types of hand drawings (i.e., the back of the left or right hand with all five fingers raised) were displayed in one of three different orientations (0°, 90° and 270°). In the character rotation task, two capital characters (i.e., G and P) were presented in the normal or the mirror-reversed form in the same three orientations. In all, six (3 × 2) images for the HLJ task and 12 (3 × 2 × 2) images for the character rotation task were prepared, respectively. Each of the 6 stimuli in the HLJ task was presented eight times, and each stimulus of the 12 in the character rotation task was presented four times. Therefore, each participant performed a total of 96 trials during the experiment. In both tasks, the stimuli were large, approximately 10.5 cm along the widest axis. The hand and character stimuli were arranged into two blocks and the block orders were counterbalanced across participants. The trials were randomized within each task. Prior to each task, participants practiced each of the six types of stimuli in the HLJ task and each of the 12 types of stimuli in the character task.

### Procedure

The participants were tested individually. The stimuli were displayed on a 32-inch monitor (Mitsubishi Co., Japan). The distance between the screen and participants was 120 cm. The stimulus presentation and the RT measurement were controlled by a computer with SuperLab 4.5 software (Cedrus Corporation, 2006). Each trial began with a fixation period (800 ms) with a black cross displayed on a white monitor, followed by a hand or character stimulus. The stimulus disappeared when a participant pressed a foot pedal. The RT (in ms) was recorded from the time when the stimulus appeared on the monitor until the participant pressed the foot pedal. The participant pressed the “left” pedal with the left foot if the displayed hand figure was the “left” hand or normal character, and pressed the “right” pedal with the right foot if the hand figure was the “right” hand or mirror character. The inter trial interval (ITI) was 2 s. During the experiment, in order to prevent the participants from comparing the stimuli with their own hands, the participants were instructed to rest their hands on their knees under the table.

**Fig 3:**
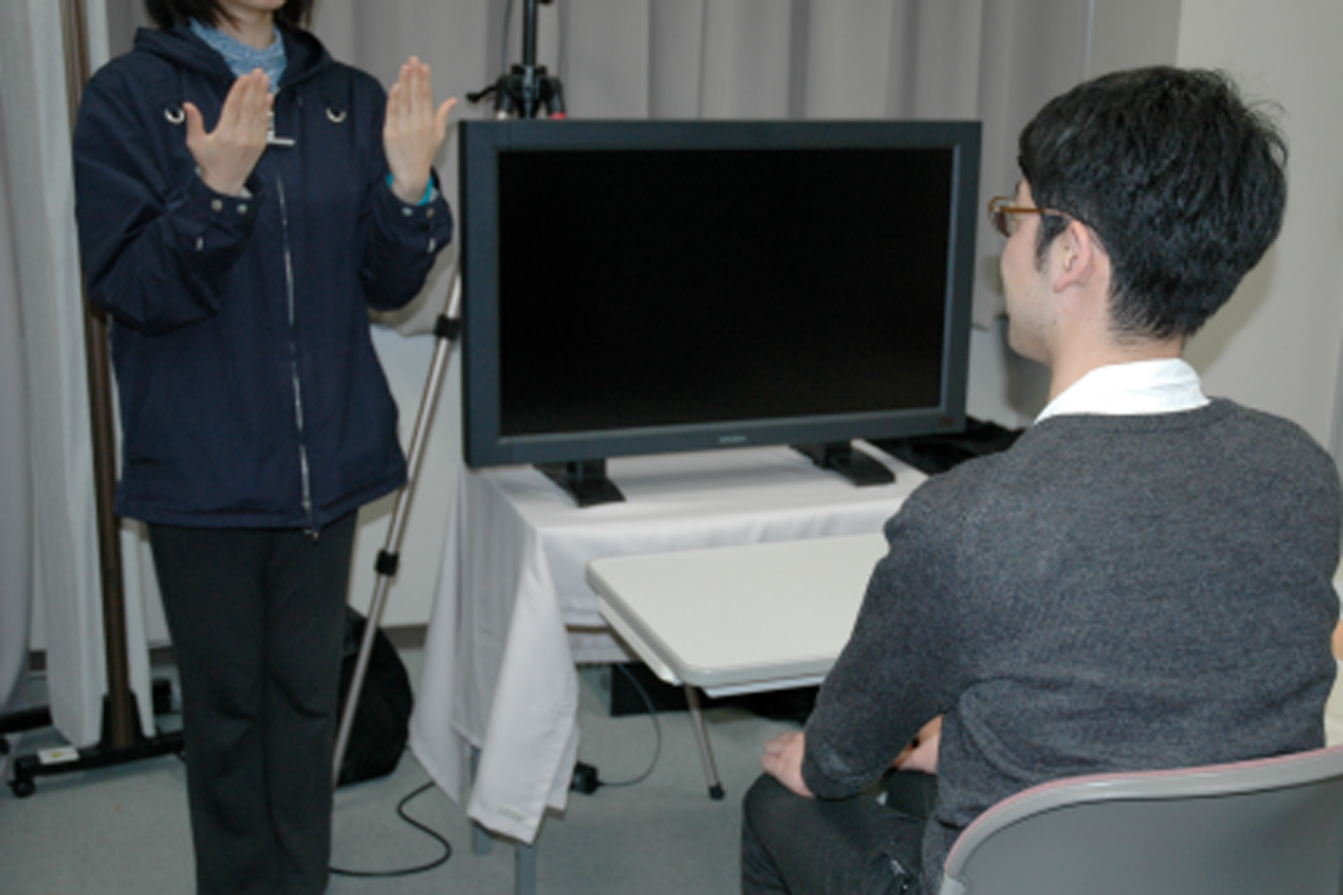
A scene of the instruction and hand demonstration to a facing participant by an experimenter in the 3PP condition.

In the 1PP condition, the participants were instructed to judge whether a hand stimulus was their left or right hand. In contrast, in the 3PP condition participants were asked to judge whether a displayed hand was another person’s (in this case the experimenter’s) left or right hand. The experimenter stood beside a monitor on which the experimental stimuli (back hand view) were displayed, face to face with the participant. The experimenter gave the instruction for the 3PP condition by showing his/her own back of the hand to the participant who sat on a chair and said, “Please imagine that the experimenter’s left or right hand is presented on a monitor, and your task is using it to decide whether the presented hand is the experimenter’s left or right hand”. It is noteworthy that the demonstration of a *back* hand view by an experimenter to a participant implies the demonstration of a *palm* hand view from the view of the experimenter in the instruction for the 3PP condition. Fig 3 is a scene of the instruction and hand demonstration to a facing participant by an experimenter in the 3PP condition.

In both conditions, the participants were asked to judge whether the character displayed was a mirror or a normal character. The hand task is the experimental task, while the character task is the control task. The order of the hand and character tasks was counterbalanced across participants.

### Apparatus

The present study used a 2-channel NIRS unit (Pocket NIRS, Dyna Sense Inc., Japan) to measure the participants’ concentration changes of oxygenated hemoglobin (Coxy-Hb), deoxygenated hemoglobin (deoxy-Hb), and total hemoglobin. Two probes were attached to the forehead using double-sided adhesive sheets and were centered on the Fp1 and Fp2 positions, respectively (according to the international 10–20 system for electroencephalogram recording). Each probe was composed of one emitter optode and one detector optode located 3 cm apart. Based on the 3-dimensional probabilistic anatomical cranio-cerebral correlation [27], Fp1 and Fp2 were projected onto the left and the right prefrontal region, and it has been shown that the NIRS detects hemodynamic changes in the brain to a depth of approximately 3 cm from the surface [28]. Thus, we assumed that the Pocket NIRS approximately measured the activation in the bilateral PFC located in close proximity to scalp tissues [22]. The sampling rate was 10 Hz.

### Behavioral data analysis

The participants of 1PP (N = 63) and 3PP (N = 37) groups were divided into two subgroups on the basis of a median split derived from the mean error rate of the hand task averaged across rotation angle, respectively [23]. Consequently, the sixty-three participants in the 1PP group was subdivided into the EG (lower performers: N = 22; error rate = 3.50%) and the NEG (higher performers: N = 41; error rate = 0). The thirty-seven participants in the 3PP group were also subdivided into the EG (N = 11; error rate = 4.36%) and the NEG (N = 26; error rate = 0). Trials with error responses and trials in which the RTs were more than 3 SDs from a participant’s overall mean were excluded from further analysis. Precisely, the following percentages of the raw data in each subgroup were discarded from further analysis. In the HLJ task, 1PP group (4.92% of the EG, 2.03% of the NEG) and 3PP group (6.44% of the EG, 1.52% of the NEG); in the character task, 1PP group (8.51% of the EG, 5.38% of the NEG) and 3PP group (11.36% of the EG, 5.93% of the NEG). The discarded raw data (error and over 3 SDs responses) in the character task was relatively higher than that in the HLJ task. Specifically, the percentage of EG in the 3PP condition showed the highest value (11.36%) in spite of the fact that the character task has no direct relation with the subgrouping (e.g., EG) based on error performance in the HLJ task and the 3PP condition used in the HLJ task. The implication will be discussed later. The remaining correct data were statistically analyzed by SPSS (Statistical Packages for Social Sciences, version 22, IBM, Tokyo, Japan).

Conson et al. [29] demonstrated that Hand (left and right) × Orientation (90° and 270°) interaction is an index of BC effect in the HLJ task. In order to test the BC effect, we followed up their data analysis. First, to investigate the effect of Performance level (higher vs. lower performers: NEG vs. EG), we separately conducted a 3-way ANOVA of the RTs, with the Performance level (NEG vs. EG) as the between factor, and the Hand Laterality (left vs. right) and Orientation (90° vs. 270°) as the two within-subject factors. Second, to investigate the effect of Perspective (1PP vs. 3PP), we separately conducted a 3-way ANOVA of the RTs, with Perspective (1PP vs. 3PP) as the between factor, and the Hand Laterality (left vs. right) and Orientation (90° vs. 270°) as the two within-subject factors. Finally, we specifically tested the BC effect by means of a 2-way ANOVA with Hand Laterality (left vs. right) and Orientation (90° or 270°) as within factors in each performance level of each perspective condition. In the character task, the same analyses were conducted. The alpha-level of all the behavioral analyses was set at *p* < .05.

### NIRS data analysis

The NIRS data of trials with error responses and trials in which the RTs were more than 3 SDs from a participant’s overall mean were excluded from further analysis. We focused on the Coxy-Hb during the tasks because oxy-Hb is the most sensitive parameter of regional cerebral blood flow [30]. The average Coxy-Hb for each channel was obtained for four measurement periods for each trial: (1) pretest (rest) period as a base line (1 s: the 2nd half of ITI 2s), (2) fixation period (0.8 s), and (3) test period (target presentation), (4) posttest (rest) period (1 s: the 1st half of ITI 2 s).

The NIRS data were converted to z-scores for the analysis [31]. The z-scores were calculated using the mean value and the standard deviation of the Coxy-Hb during the (1) pretest period as a baseline for the (3) test period in each trial as in the manner of an event-related paradigm. The z-scores were averaged over trials for each stimulus type (hand: left and right; character: normal and mirror). Thus, we finally obtained group-averaged z-scores for each stimulus type for each angle. The average values of the z-scores for Coxy-Hb during the (3) test and (4) posttest periods were used in the analysis.

## Results

### Reaction time in the HLJ task

#### Performance level effect (higher vs. lower performers: NEG vs. EG)

We subdivided the participants in both the 1PP and 3PP groups into NEG and EG according to their error rates in the HLJ task, which accounted for 0% response in the NEG and for 3.93% in the EG. Because of these high accuracy levels, statistical analysis was performed on correct RTs only. We conducted sequentially three types of ANOVA to test the following three effects: (a) Performance level effect (higher vs. lower performers: NEG vs. EG), (b) Perspective effect (1PP vs. 3PP), and (c) BC effect (reversed BC to left and right hands: medial vs. lateral rotations). First, we will report the RT profiles of the HLJ task and character task, and second the NIRS profiles of the two tasks.

**Fig 4:**
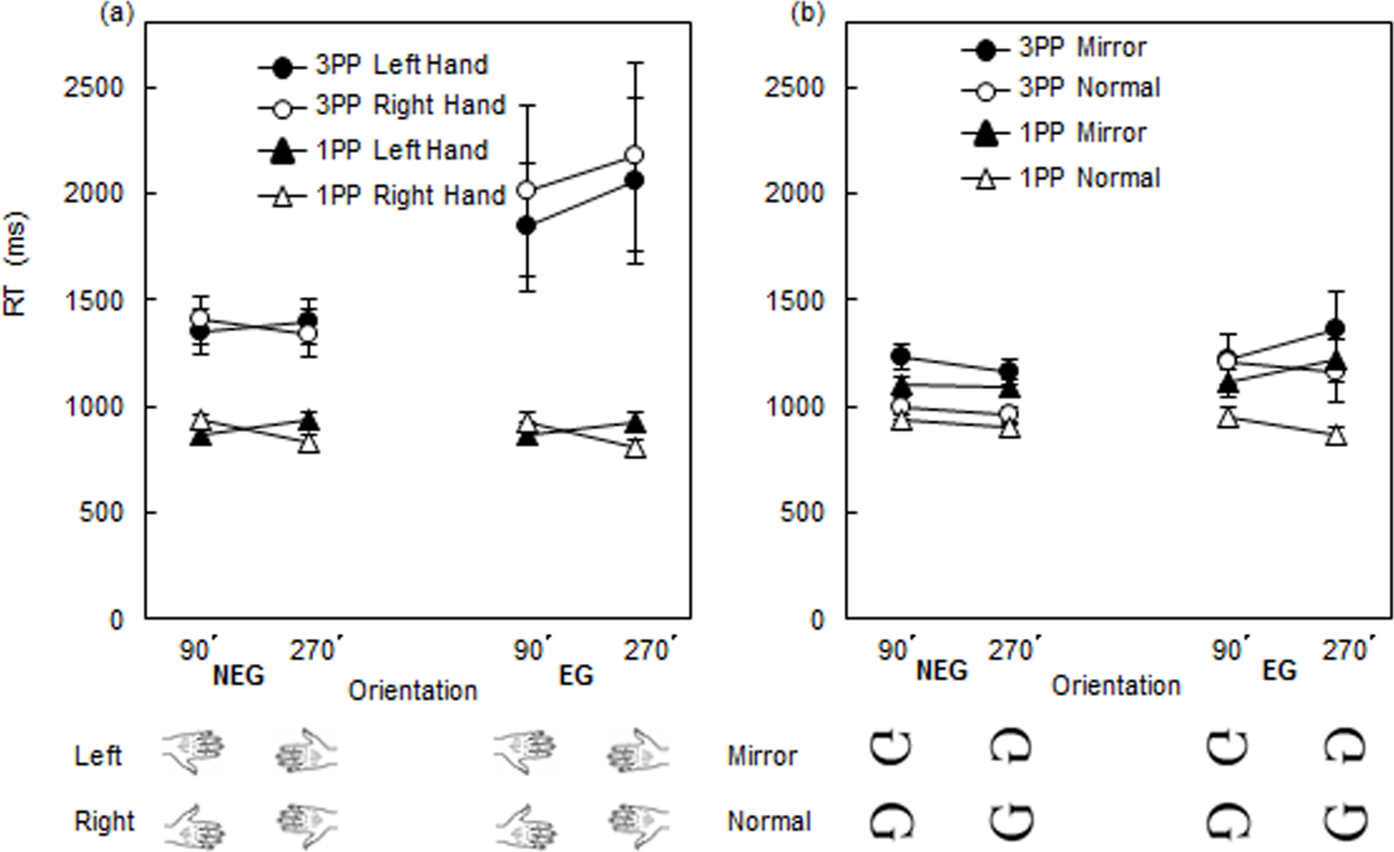
RT (Mean ± standard error of the mean (M ± SEM)) of the EG and NEG in the 1PP and the 3PP conditions of (a) the hand task and (b) the character task at 90° and 270°.

Fig 4(A) depicts the RT of the NEG and EG in the 1PP and the 3PP conditions in the HLJ task. In order to test the effect of Performance levels (NEG vs. EG) on RT, we conducted a 3-way mixed ANOVA for the 1PP and 3PP, separately, with the Performance level (NEG, EG) as the between-subject factor and with Hand (left, right) and Orientation (90°, 270°) as the two within-subject factors.

In the 3PP condition, the results revealed that the main effect of Performance level was significant (*F*(1,35) = 5.15, *p* < .05, η^2^ = 0.13) due to the fact that the mean RT was significantly longer for the EG (1972.89 ms) than that for the NEG (1317.63 ms), and the main effect of Orientation was significant (*F*(1,35) = 4.76, *p* < .05, η^2^ = 0.12). A first-order interaction of Performance level × Orientation was significant (*F*(1,35) = 5.81, *p* < .05, η^2^ = 0.14). No other main effect or interactions were significant.

In the 1PP condition, the results revealed that the main effect of Orientation was significant (*F*(1,61) = 4.55, *p* < .05, η^2^ = 0.07). A first-order interaction of Hand × Orientation was significant (*F*(1,61) = 24.53, *p* < .001, η^2^ = 0.29). No other main effects or interactions were significant.

#### Perspective effect (1PP vs. 3PP)

In order to examine the effect of Perspectives, we conducted a 3-way mixed ANOVA for the NEG and EG, separately, with Perspective as the between-subject factor and Hand and Orientation as the two within-subject factors. In the NEG, the results revealed that the main effect of Perspective was significant (*F*(1, 65) = 29.35, *p* < .001, η^2^ = 0.31) due to the fact that the mean RT was significantly longer for the 3PP (1317.63 ms) than that for the 1PP (856.76 ms). A first-order interaction of Hand × Orientation was significant (*F*(1, 65) = 14.98, *p* < .001, η^2^ = 0.19). No other main effects or interactions were significant (see, Fig 4(A)).

In the EG, the results revealed that the main effect of Perspective was significant (*F*(1, 31) = 18.92, *p* < .001, η^2^ = 0.38) due to the fact that the mean RT was significantly longer for the 3PP (1972.89 ms) than that for the 1PP (832.81 ms), and the main effect of Orientation was significant (*F*(1,31) = 5.85, *p* < .05, η^2^ = 0.16). A first-order interaction of Perspective × Orientation was significant (*F*(1,31) = 12.04, *p* < .01, η^2^ = 0.28). There were no other main effects or significant interactions (see, Fig 4(A)).

#### BC effect (Reversed BC to left and right hands: medial vs. lateral rotations)

In order to investigate the BC effect, we specifically conducted a 2-way repeated measures ANOVA with Hand and Orientation for four subgroups (NEG and EG both in 1PP and 3PP conditions), separately. In the NEG of 1PP condition, the results showed that the main effects of Hand and Orientation were not significant, but the Hand × Orientation interaction was significant in the NEG of the 1PP condition (*F*(1, 40) = 19.71, *p* < 0.001, η^2^ = 0.33). Simple main effect tests revealed significant differences: the RT of the right hand (933.80 ms) was longer than that of the left hand (869.72 ms) at 90° (*F*(1, 40) = 7.36, *p* < .05, η^2^ = 0.16), and the RT of the left hand (941.34 ms) was longer than that of the right hand (836.83 ms) at 270° (*F*(1, 40) = 17.19, *p* < .001, η^2^ = 0.30). The RT of the left hand (941. 34 ms) at 270° was longer than that of the left hand (869.72 ms) at 90° (*F*(1, 40) = 9.35, *p* < .01, η^2^ = 0.19); analogously, the RT of the right hand (933.80 ms) at 90° was longer than that the right hand (836.83 ms) at 270° (*F*(1, 40) = 22.60, *p* < .001, η^2^ = 0.37).

Similarly, in the EG of the 1PP condition, there was neither a main effect of Hand nor a main effect of Orientation, but there was a significant interaction of Hand × Orientation (*F*(1,21) = 7.81, *p* < .05, η^2^ = 0.27). Simple main effect tests revealed that the RT of the right hand (924.90 ms) at 90° was significantly longer than that of the right hand (807.17ms) at 270° (*F*(1, 21) = 8.39, *p* < .01, η^2^ = 0.29), and the RT of the left hand (922.58 ms) was significantly longer than that of the right hand (807.17 ms) at 270° (*F*(1, 21) = 9.02, *p* < .01, η^2^ = 0.30).

While in the 3PP condition, except for a significant main effect of Orientation in the EG (*F*(1,10) = 5.43, *p* < .05, η^2^ = 0.35), none of the remaining main effects or interactions were significant in either the EG or the NEG.

### Character task

#### Performance level effect (NEG vs. EG)

Fig 4(B) depicts the RT of the EG and NEG in the 1PP and the 3PP conditions in the character task. We conducted a 3-way mixed ANOVA for the 1PP and 3PP, separately, with Performance level (NEG vs. EG) as the between-subject factor and with the Character (mirror, normal) and Orientation (90°, 270°) as the two within-subject factors. In the 3PP condition, the results revealed that the main effect of Character was significant (*F*(1, 35) = 20.56, *p* < .001, η^2^ = 0.37). There were no other main effects or significant interactions.

In the 1PP condition, the results revealed that the main effect of Character was significant (*F*(1, 61) = 74.90, *p* < .001, η^2^ = 0.55). A first-order interaction of Character × Orientation was significant (*F*(1,61) = 11.65, *p* < .01, η^2^ = 0.16), and a second-order interaction of Performance × Character × Orientation was significant (*F*(1, 61) = 6.87, *p* < .05, η^2^ = 0.10). No other main effects or interactions were significant.

#### Perspective effect (1PP vs. 3PP)

We conducted a 3-way mixed ANOVA for the NEG and EG, separately, with the Perspective as the between-subject factor and with the Character and Orientation as the two within-subject factors. In the NEG, the results revealed that the main effect of Character was significant (*F*(1,65) = 120.45, *p* < .001, η^2^ = 0.65), and the main effect of Orientation was significant (*F*(1,65) = 7.85, *p* < .01, η^2^ = 0.11). There were no other main effects or significant interactions. (see, Fig 4(B)).

In the EG, the results revealed that the main effect of Character was significant (*F*(1,31) = 13.34, *p* < .01, η^2^ = 0.30). A first-order interaction of Character × Orientation was significant (*F*(1, 31) = 7.61, *p* < .01, η^2^ = 0.20). No other main effects or interactions were significant (see, Fig 4(B)).

#### BC effect

We conducted a 2-way repeated measures ANOVA with Character and Orientation for four subgroups (NEG and EG in both the 1PP and 3PP conditions), separately. The RT profiles of EG and NEG in the 1PP condition and of EG in the 3PP condition showed the following significant (main effect of Orientation), respectively: EG in 1PP: *F*(1, 21) = 22.83, *p* < .001, η^2^ = 0.52; NEG in 1PP: *F*(1, 40) = 59.73, *p* < .001, η^2^ = 0.60; EG in 3PP *F*(1, 25) = 65.44, *p* < .001, η^2^ = 0.72. The results showed that the mirror characters (MC) required a significantly longer RT than that of the normal characters (NC) in the EG and NEG in the 1PP condition (MC: 1166.87 ms > NC: 908.44 ms; MC: 1099.81 ms > NC: 920.19 ms, respectively), and also in the NEG in the 3PP condition (MC: 1195.70 ms > NC: 977.51 ms) (see, Fig 4(B)). The EG in the 3PP condition did not show the main effect of MC vs. NC (MC: 1293.43 ms; NC: 1187.57 ms); however, the interaction between the Character and the Orientation was significant in the EG in the 1PP condition (*F*(1, 21) = 8.32, *p* < .01, η^2^ = 0.28). Simple main effect tests revealed that the RT of normal characters at 90° (954.60 ms) was significantly longer than that of normal characters at 270° (862.24 ms) (*F*(1, 21) = 18.35, *p* < .001, η^2^ = 0.47).

### NIRS data in the HLJ task

#### Performance level effect (NEG vs. EG)

**Fig 5:**
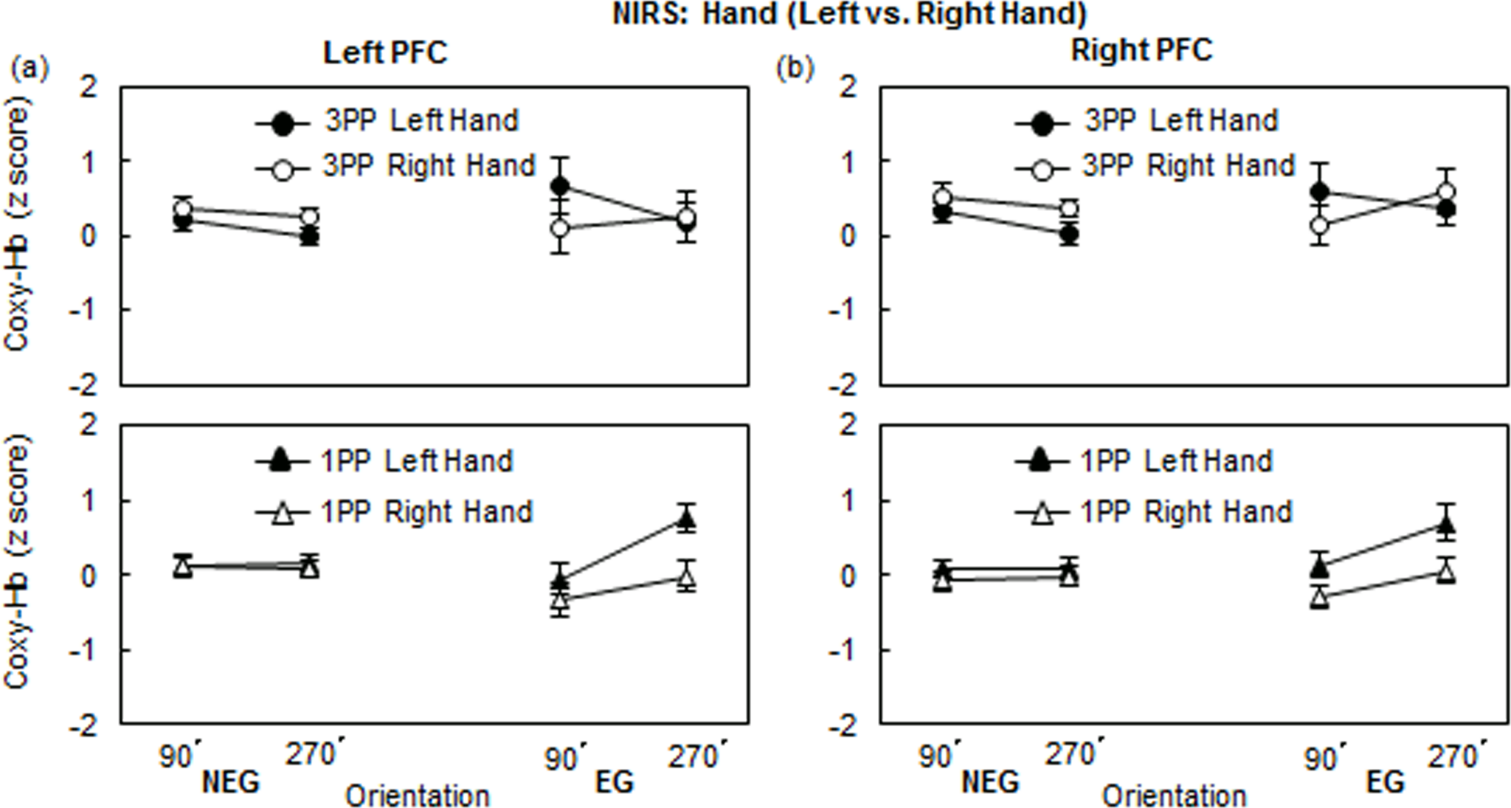
Coxy-Hb changes (M ± SEM) of the EG and NEG in the 1PP and the 3PP conditions of (a) left PFC and (b) right PFC in the hand task at 90° and 270°.

Focusing on the NIRS profiles of Left and Right hands, Fig 5 illustrates the z-scores for Coxy-Hb of the EG and NEG in the 1PP and the 3PP conditions in the HLJ task. We conducted a 3-way mixed ANOVA for the 1PP and 3PP, separately, with Performance level (NEG vs. EG) as the between-subject factor and with the Hand (left, right) and Orientation (90°, 270°) as the two within-subject factors. The analyses were conducted on the averaged value of the z-scores for Coxy-Hb during the test and posttest periods. In the 3PP condition, no main effects or interactions were significant (see, Fig 5).

In the 1PP condition, the left PFC activation showed that the main effect of Hand was significant (*F*(1,61) = 6.59, *p* < .05, η^2^ = 0.10). A first-order interaction of Performance level × Hand was significant (*F*(1,61) = 4.40, *p* < .05, η^2^ = 0.07), and a first-order interaction of Performance level × Orientation was significant (*F*(1,61) = 4.25, *p* < .05, η^2^ = 0.07. No other main effect or interaction was significant (see, Fig 5(A)). The right PFC activation showed that the main effect of Hand was significant (*F*(1,61) = 7.54, *p* < .01, η^2^ = 0.11), and the main effect of Orientation was significant (*F*(1,61) = 4.83, *p* < .05, η^2^ = 0.07). A first-order interaction of Performance level × Hand was significant (*F*(1,61) = 2.78, *p* = .10, η^2^ = 0.04) There were no other main effects or significant interactions (see, Fig 5(B)).

#### Perspective effect (1PP vs. 3PP)

**Fig 6:**
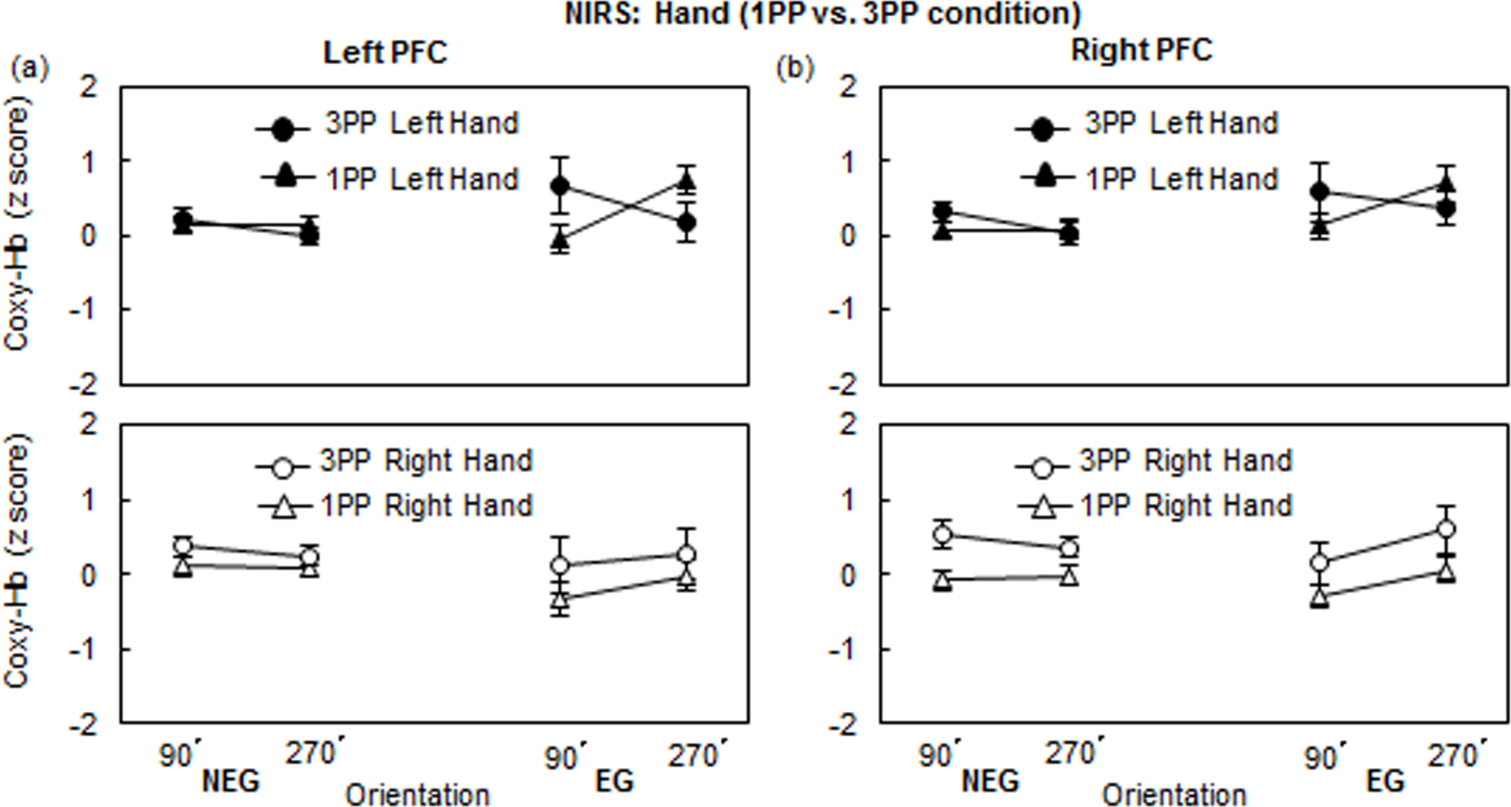
Coxy-Hb changes (M ± SEM) of the 1PP and 3PP conditions (a) left PFC and (b) right PFC in the hand task at 90° and 270°.

Focusing on the NIRS the profiles of 3PP and 1PP, Fig 6 illustrates the z-scores for Coxy-Hb changes of the 1PP and 3PP conditions in the HLJ task. We conducted a 3-way ANOVA, with Perspective as the between-subject factor and the Hand and Orientation as the two within-subject factors. In the NEG, the left PFC activation showed no main effects or interactions. The right PFC activation showed that the main effect of Perspective was significant (*F*(1, 65) = 8.57, *p* < .01, η^2^ = 0.12). No other main effects or interactions were significant. In the EG, no main effects or interactions were significant (see, Fig 6).

#### BC effect

We conducted a 2-way repeated measures ANOVA with Hand and Orientation for four subgroups (NEG and EG both in 1PP and 3PP conditions), separately. The results showed that the bilateral PFC showed significantly higher activations when a left hand picture was presented, as compared to when a right hand picture was presented (Left PFC: *F*(1,21) = 7.94, *p* < .05, η^2^ = 0.28; Right PFC: *F*(1,21) = 8.32, *p* < .05, η^2^ = 0.28) in the EG in the 1PP condition (Fig 5). However, none of the interactions were significant. In contrast, in the 3PP condition, none of the main effects were significant in either the EG or the NEG, but the activity of the left PFC of the EG showed an interaction of Hand × Orientation (*F*(1,10) = 6.59, *p* < .05, η^2^ = 0.40), while the activity of the right PFC of the EG did not show the significant interaction (Fig 5).

In order to determine whether the brain activities of 1PP and 3PP conditions are mirror-reversed or not, we performed a two-way mixed ANOVA with Orientation as the within factor and Group as the between factor. In the EG, when a *left* hand picture was presented, the activations of *left* PFC in the 1PP and 3PP conditions were significantly reversed (Orientation × Group: (*F*(1,31) = 6.03, *p* < .05, η^2^ = 0.16) (Fig 6(A)); although the activation of the *right* PFC in the 1PP and 3PP conditions were mirror-reversed, but the interaction was not significant (Fig 6(B)).

### Character task

#### Performance level effect (NEG vs. EG)

**Fig 7:**
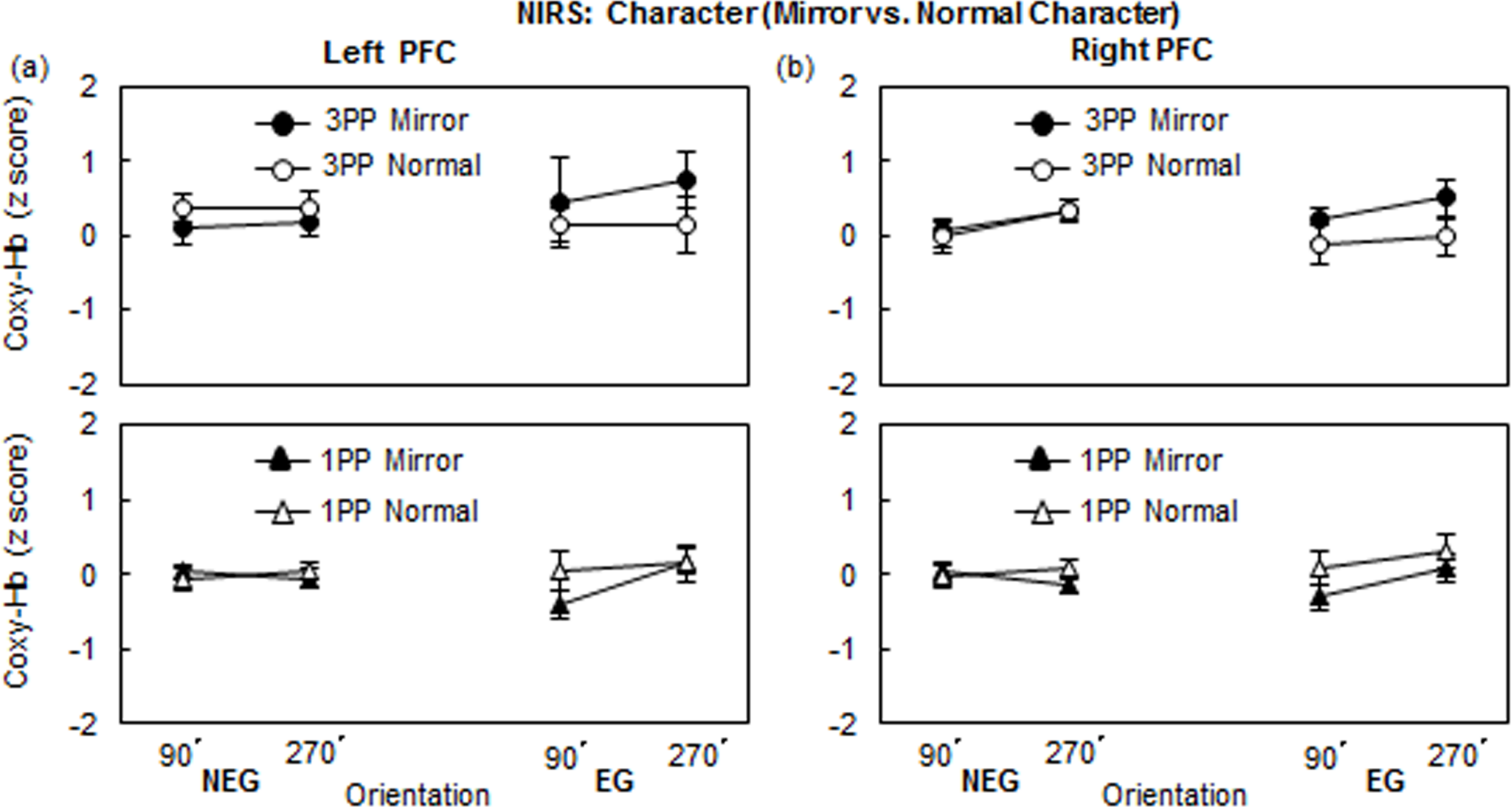
Coxy-Hb changes (M ± SEM) of the EG and NEG in the 1PP and the 3PP conditions of (a) left PFC and (b) right PFC in the Character task at 90° and 270°.

Focusing on the NIRS profiles of Mirror and Normal characters, Fig 7 illustrates the z-scores for Coxy-Hb of the EG and NEG in the 1PP and the 3PP conditions in the Character task. We conducted a 3-way mixed ANOVA for the 1PP and 3PP, separately, with Performance level (NEG, EG) as the between-subject factor and with the Character (mirror, normal) and Orientation (90°, 270°) as the two within-subject factors. In neither the 1PP nor the 3PP condition, were any main effects or interactions significant.

#### Perspective effect (1PP vs. 3PP)

**Fig 8:**
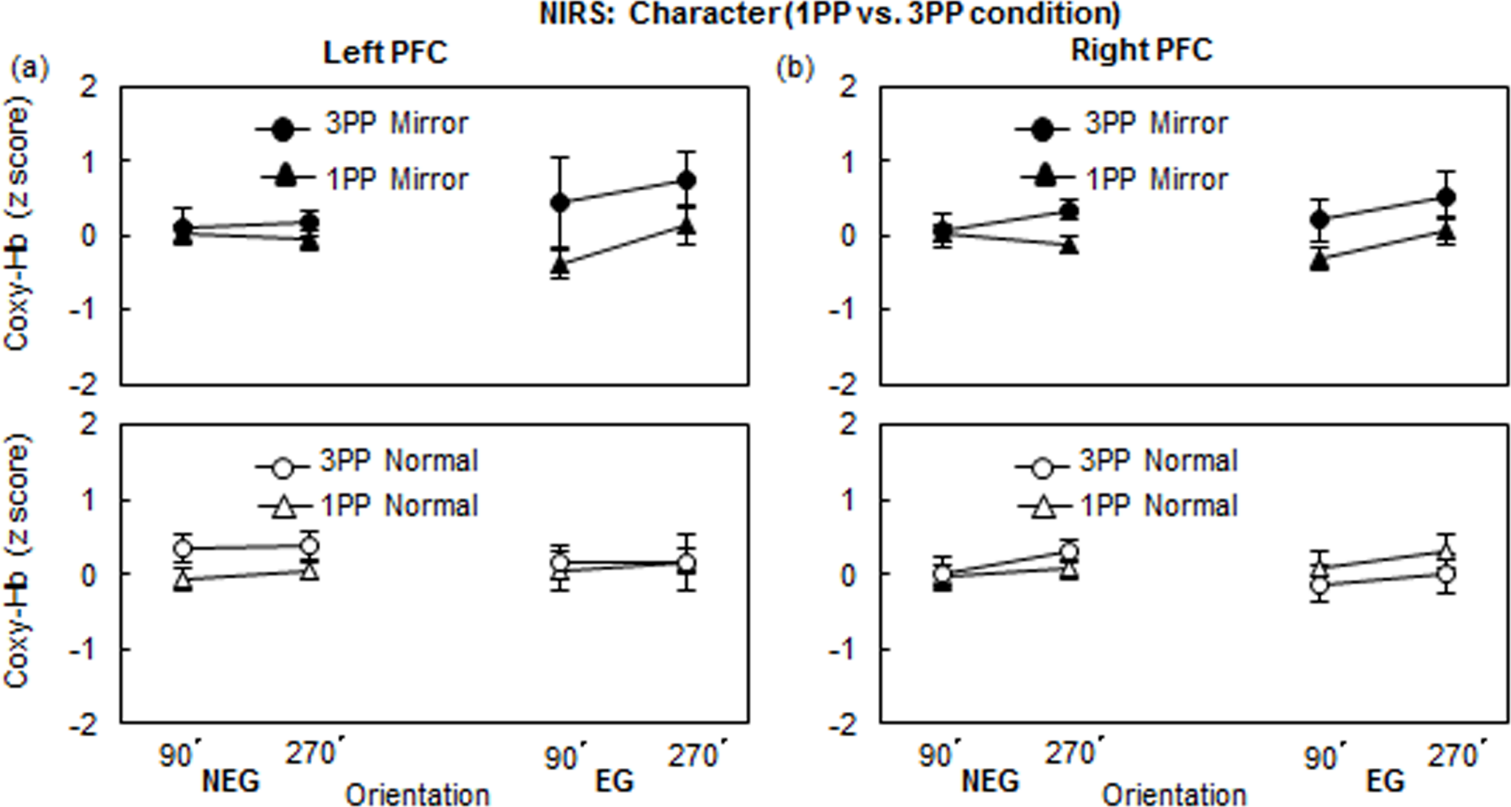
Coxy-Hb changes (M ± SEM) of the 1PP and 3PP conditions (a) left PFC and (b) right PFC in the Character task at 90° and 270°.

Focusing on the NIRS the profiles of 3PP and 1PP, Fig 8 illustrates the z-scores for Coxy-Hb changes of the 1PP and 3PP conditions in the Character task. We conducted a 3-way ANOVA, with Perspective as the between-subject factor and the Character and Orientation as the two within-subject factors. In the NEG, the left PFC activation showed that the main effect of Perspective was significant (*F*(1, 65) = 4.92, *p* < .05, η^2^ = 0.07. There were no other main effects or significant interactions (see, Fig 8). In the right PFC of EG and bilateral PFC of NEG, no main effects and interactions were significant.

#### BC effect

We conducted a 2-way repeated measures ANOVA with Character and Orientation for four subgroups (NEG and EG both in 1PP and 3PP conditions), separately. The results showed that there were no significant main effects or interactions in bilateral PFC (Fig 8). Furthermore, to determine whether the brain activities of 1PP and 3PP conditions are mirror-reversed or not, we conducted a two-way mixed ANOVA with Orientation as the within factor and Group as the between factor. In the EG, the results showed that the brain activations of 1PP and 3PP are not mirror-reversed, but the mirror characters in the 3PP condition triggered significantly larger brain activities than that in the 1PP condition (Left PFC: (*F*(1,31) = 5.12, *p* < .05, η^2^ = 0.14; Right PFC: (*F*(1,31) = 6.05, *p* < .05, η^2^ = 0.16) (Fig 8).

## Discussion

In the present study, we investigated the BC effect in the HLJ task from 1PP and 3PP. To achieve the goal, we explicitly instructed the 1PP group to judge whether the presented hand was their *own* left or right hand, and the 3PP group to judge whether it was the *others*’ left or right hand. Although the 37 participants in the 3PP condition were explicitly instructed to take a 3PP, they could take a 1PP to achieve the HLJ task in the 3PP condition. Thus, we separated the 37 participants in the 3PP condition into the NEG (26 higher performers: 70%) and EG (11 lower performers: 30%) according to their error rates in the HLJ task. Presumably, the NEG might take a Back-Back strategy and the EG a Back-Palm strategy. In the same manner, we also divided the 63 participants in the 1PP condition into the NEG (41 higher performers: 65%) and EG (22 lower performers: 35%). Consequently, subgrouping on the basis of the error rates in the HLJ task showed that the NEG in both the 1PP and 3PP had literally zero errors, while the EG in both the 1PP and 3PP showed errors, i.e., 3.50% and 4.36%, respectively. We hypothesized that if the EG in the 3PP condition, might strictly follow the 3PP instruction and take a Back-Palm strategy, the profile of their PFC activation would show the BC effect in the HLJ task as well as the EG in the 1PP group.

Before discussing the main profile in the HLJ task, we assess the profile in the character task conducted as a control condition for the HLJ task. It is worthy to note that NEG and EG were separated on the basis of their error rates in the *HLJ task*, but not in the *character task*. Nevertheless, it is interesting that the error rates of the EG both in 1PP and 3PP conditions (1PP, 6.44%; 3PP, 9.47%) in the character task were relatively higher than those of the NEG (1PP, 3.66%; 3PP, 4.01%). These results may imply that the participants in the NEG are more adept in the mental rotation task of hand (body) and character (object) regardless of the stimulus types. The adeptness, however, may imply a higher ability of mental rotation and also a talent for problem solving such as awareness of the orientation of thumb up and thumb down.

The RT profiles in the character task did not show an interaction between Mirror/Normal and Orientation except the EG in the 1PP condition. The exceptional interaction of EG is that the RTs for mirror characters were longer than those for normal characters both at 90° and 270°, while the RTs for normal characters at 270° were shorter than those at 90°. In addition, the error rate for normal characters at 270° was lower than that at 90°. According to a post hoc analysis for the character types in the character task, the character “G” showed the interaction at mirror 270° and normal 90°. These two patterns of G look similar and may be hard to identify due to the font type and spatial resolution of the character G. Furthermore, none of the NIRS profiles showed any interaction between Mirror/Normal and Orientation obtained for RT in the character task. Therefore, in the character task, the exceptional interaction for RT of EG in the 1PP condition was not caused by the BC. The reliable results of the EG in the character task are the following two: the RT in the 3PP condition was longer than that in the 1PP condition, and correspondently, the PFC activation in the 3PP condition was higher than that in the 1PP condition, especially for mirror judgment.

Although the participants in both the 1PP and 3PP groups received the same instruction for the character task, the PFC activation of the EG in the 3PP condition was higher than that in the 1PP condition (see Fig 8). The result of the higher PFC activation of the EG in the 3PP to the character task can be interpreted by a *spill over effect* from the HLJ task: the 3PP group was instructed to judge presented hands from the other person’s perspective. The task demand of taking the view of the other person (3PP) requires reversing one’s *own* hand image (palm or back) into the *other’*s hand, vice versa. We speculate that the reverse view of the HLJ task in the 3PP might induce a higher cognitive load to judge the mirror image of the character, because if the EG viewed the displayed mirror character from the back side, EG might confuse the mirror character with the normal character. The speculation of a spill over effect on the character task was confirmed by a post hoc analysis of RT of EG in the 3PP condition, but not in the 1PP condition. To accept this interpretation, sufficient evidence with statistical validation is needed in future studies.

In the HLJ task, the RT profiles of both NEG and EG varied with BC in the 1PP conditions. These results on RT are consistent with the previous studies [6–9], which indicate that participants in the 1PP condition imagine moving their *own* hand to perform the task. In contrast to the 1PP condition, in the 3PP condition, the RTs in neither the EG nor the NEG showed the significant interactions between Hand and Orientation obtained in the 1PP condition. The RT profile of the NEG in the 3PP condition showed a similar pattern of the interaction in the 1PP condition, although it was not statistically significant.

We confirmed that RTs profiles in the 3PP condition were longer than that those in the 1PP condition, which is consistent with previous studies [11, 15, 20]. The subtracted RTs between the NEG and EG in in the 3PP condition showed that the RT in the EG was significantly longer (approximately 650 ms) than that in the NEG, and the RTs of the NEG and EG in the 3PP condition were longer (approx. 500 ms and 1150 ms, respectively) than the corresponding RTs in the 1PP condition (Fig 4). These results suggest that imaging from the other’s perspective (3PP) requires a greater cognitive load for the mental operation than that from one’s own perspective (1PP).

The cognitive load in the 3PP condition may be divided into two types of mental processing, i.e., a body-image transformation and a hand-image rotation. First, the subtracted RT in NEG between 1PP and 3PP conditions ((3PP - 1PP) in NEG = 500 ms) may represent the process of mentally rotating her/his body in order to place her/himself in the opposite side with respect to their actual position. Second, the subtracted RT between NEG and EG in 3PP conditions ((EG—NEG) in 3PP = 650 ms) may represent the process of mentally rotating a hand. If we postulate a common body-image transformation between the NEG and EG in the 3PP condition, the subtracted RT ((EG—NEG) in 3PP = 650 ms) may be explained by two types of hand-image rotation, i.e., a Back-Back strategy in the NEG who always keep a back hand image, and a Back-Palm strategy in the EG who obtain the *other’s palm* hand image from one’s *own back* hand image.

These RT profiles in the present study may support a hypothetical model of hand representation for a 3PP in which the mental operations in the 3PP condition consist of two stages of a body-image transformation and a hand-image rotation. The hand-image rotation diverges in a relatively parsimonious Back-Back strategy and multiaxial Back-Palm strategy. The multiaxial hand-image rotation might be used to confirm the vagueness of a final rotated hand-image. This type of confirmation for body movement by imaging goal behavior is commonly used in sports as an imaging rehearsal (i.e., image training) for a relevant mulitiaxial-behavior.

Although the RT in the NEG showed a similar interaction pattern in the 3PP condition, it did not reach statistical significance. In addition, the NEG in the 3PP condition might have taken a 1PP using a Back-Back strategy, leading to shorter RTs than the EG who took a Back-Palm strategy in the 3PP condition, and to longer RTs than the NEG in the 1PP condition due to the additional body rotation in the 3PP condition.

On the other hand, although the RT profile of EG in the 1PP condition showed the BC effect (a Hand × Orientation interaction), the RT profile of EG in the 3PP condition did not show the BC effect with a reversed RT pattern against the BC effect in the 1PP condition. However, the reversed interaction was partially revealed when we focused on the RT profile of the right hand in the EG between the 1PP and 3PP conditions. That is, the EG in 1PP showed an increasing RT from 270° (medial rotation) to 90° (lateral rotation), while the EG in 3PP showed an increasing RT from 90° (medial rotation) to 270° (lateral rotation) if they adopt the palm hand view. The most important point is that the reversed increasing pattern of RTs for the right hand between 1PP and 3PP can be explained by the back hand view in 1PP and the palm hand view in 3PP, which is consistent with a reversed view point of lateral and medial rotations. However, RT for the left hand in 1PP increased from 90° (medial rotation) to 270° (lateral rotation), but the RT for the left hand in 3PP did not increase from 270° to 90°. One possible interpretation is that the processing of mental rotation for the left hand in 3PP might be entrained to the processing of a dexterous right hand which is more easily processed than the awkward left hand. The tentative interpretation for the reversed interaction hypothesis is based on the RT profiles, but the larger standard deviation of EG in 3PP on RT makes it hard to directly compare RT across the four subgroups (1PP/3PP × EG/NEG). This controversial hypothesis must be examined in terms of the PFC activation of EG in the 1PP and 3PP conditions for the *left* hand stimuli.

The left PFC activation of EG in the 3PP condition a significant interaction between Hand and Orientation (see Fig 5(A)), while the NIRS profile of NEG did not show the interaction (see Fig 5(A)). Precisely, for a Back-Palm strategy (see Fig 2), the left PFC activation of EG in the 3PP condition consisted of higher activation at 90° (*lateral* rotation) than at 270° (*medial* rotation) for the *left* hand stimulus, and a slightly higher activation at 270° (*lateral*) than at 90° (*medial*) for the *right* hand stimulus. Whereas the left PFC of EG activation in the 1PP condition showed a higher activation at 270° (*lateral*) than at 90° (*medial*) for the *left* hand from a back hand view, but it did not show a higher activation at 90° (*lateral*) than at 270° (*medial*) for the *right* hand. Taken together, we confirmed the significant interaction between Perspective (1PP vs. 3PP) and Orientation (90° vs. 270°) for left hand stimuli in the *left* PFC of lower performers (EG) (Fig 6(A)). The PFC activation patterns in the right hemisphere were similar to those in the left hemisphere, but did not show a significant interaction (Fig 6(B)). The clear BC effect for the *left* hand stimuli in 1PP and 3PP conditions in the HLJ task using NIRS are same as those of a NIRS study by Meng et al. [32] who reported the left SPL activation in the 1PP condition.

These findings in the present NIRS study extend and refine previous findings on motor imagery using RT [6–9]. That is, our results of PFC activation show three prominent features, demonstrating BC effect in the 3PP condition.

First, concerning the profile of the Coxy-Hb change in the left PFC, the EG showed the Perspective (1PP vs. 3PP) × Orientation (90° vs. 270°) interaction, when the *left* hand stimulus was presented. That is, the left PFC activation for the left hand stimuli of EG in the 1PP condition (increasing from 90° to 270°) was opposite to that of EG in the 3PP condition (increasing from 270° to 90°). These results suggest that hand stimuli in the egocentric condition are automatically processed as one’s own body parts in the 1PP condition, whereas hand stimuli in the allocentric condition are processed as hands of the observer facing the participants [15]. These two processes are modulated by the BC, indicating that 1PP and 3PP share the same representation system regardless of differences of hand images (back or palm). Although our NIRS results support the idea that the PFC is involved in the amount of imagined self-rotation required during perspective taking [20], in order to precisely separate and measure specific hand and body rotations, we need a more appropriate experimental design. For instance, ideally, a systematical manipulation of imagined self-body rotation is expected during perspective transformations, which is beyond the scope of the present experimental design on imagined hand rotations.

Second, NIRS data between lateral- vs. medial- and/or in-depth vs. in-plane rotated stimuli (see Fig 2) can be used as an excellent measure to examine the engagement of motor imagery (see Figs 5(A) and 6(A)). The BC effect in the HLJ task involving more than one axis of rotation is consistent with the results of a previous study [24], suggesting that the HLJ task performed from the 3PP induces the use of motor imagery. Interestingly, it is worthy to note that NIRS detects right-handed PFC activation for left hand stimuli in the HLJ task. Brain activations can be measured using NIRS allowing participants relatively greater freedom of hand movement, even though they could not directly imitate the presented hand shape. The potential freedom of hand movement by means of NIRS in this study might aid in eliciting a shared hand representation between 1PP and 3PP. In this case, the BC effect would depend on the number of axes of rotation required for the hand stimulus.

Third, our results indicate that manipulating the instructions for the 1PP and 3PP conditions (i.e., one’s own hand in 1PP vs. the other’s hand in 3PP) may induce the perspective switching in the experiment, if the relevant participants appropriately follow their given instructions for each condition to the same set of stimuli. The crucial differences in the instructions deserve to be measured both by performance of the participants and their brain activities.

Finally, the most critical questions in the present study are summarized as follows from a discrepancy between RT and NIRS profiles. Although we obtained an interaction between Hand and Orientation on RT in the 1PP regardless of the EG and NEG, we failed to obtain the reversed interaction in the 3PP. Specifically, why did we fail to obtain the BC effect in the 3PP condition with the RT profiles of imagined others’ hand rotation in the EG? One plausible answer is that the RT concerning multiple processing in the 3PP such as body- and hand-image rotations at other person’s view varies with each individual, leading to larger standard deviations. Nevertheless, why did we obtain the BC effect in the 3PP condition with the NIRS profiles, specifically in the EG, but not in the NEG? To provide a unified answer to these questions, we need to focus on the fact that the participants’ cognitive load varies according to the task difficulty (e.g., egocentric vs. allocentric perspective taking; single- vs. multi-axis of hand rotations) and the participants’ trait (awkward-left vs. skillful-right hand) in the present study. The tentative answer is that the NIRS profiles may imply an accumulation of higher cognitive loads in the present study. That is, the left PFC activation of EG for the left hand stimuli in the present study may reflect an accumulation of higher cognitive loads induced by a task difficulty (hand-image rotation at other person’s view) and a participant trait (awkward-left hand of less skillful participants (EG)).

We interpreted the PFC activation of the EG and NEG in the 3PP condition by a Back-Palm strategy in the EG and a Back-Back strategy in the NEG. The remaining question is if the interpretation is correct, why did some participants in the EG and NEG take a different strategy for the same task demand for a 3PP? The most promising explanation is a resistance to perspective transformations, in which extra cognitive resources are required to resist against the interference from a 1PP and are needed to supply a demand for a 3PP. The idea deserves consideration from a combination of inhibition of a 1PP and facilitation of 3PP, as well as an individual proficiency of imaging ability [20, 33–34].

Additionally, as palm-view hands show a stronger BC effect than a back hand view in 1PP condition [7, 10, 32] the BC effect in 3PP condition obtained in this study using only back hand views should be tested with several different hand views. Future research is warranted using a variety of hand views, including a palm hand view, to examine this issue.

Although the results of the present study have implications for clinical applications, further data of motor-impaired individuals performing the HLJ task is needed for rehabilitation purposes for various types of patients with strokes, Parkinson’s disease, and complex regional pain syndrome. Furthermore, in the present study, only the activity of PFC was measured. PFC is involved in multiple computations, while motor related regions are relatively specified in computation of motor imagery. Future research is needed to examine the further aspects of BC in the motor related regions.

## Conclusions

The present NIRS study was designed to examine the neural correlates of biomechanical constraints (BC) in the hand laterality judgment (HLJ) task performed from a 1PP and a 3PP. To achieve the goal, we focused on the roles of body- and hand-image rotations of right handed participants and tested whether the PFC is involved in both motor imagery and perspective taking.

The NIRS profile of relatively higher error performers both in 1PP and 3PP conditions showed an interaction of Hand laterality × Orientation (biomechanical constraints effect) in their left PFC. Specifically, the left PFC activation of lower performers (EG) between 1PP and 3PP conditions showed an interaction (Perspective × Orientation) for the presented left hand stimuli. These results indicate that BC interferes with the HLJ performed from the 3PP as well as the 1PP.
